# 2023 Editors’ Highlights

**DOI:** 10.1038/s42004-023-01095-x

**Published:** 2024-01-09

## Abstract

At *Communications Chemistry* we are pleased to launch an Editors’ Highlights Collection featuring some of our favourite articles published in the journal in 2023. Here, we highlight each article and outline why it was selected.

Senior Editor Dr Teresa Schauperl highlights *Depolymerization of robust polyetheretherketone to regenerate monomer units using sulfur reagents* by Yasunori Minami et al. (10.1038/s42004-023-00814-8). “The work is part of a Guest Edited Collection on the topic of organomediated polymerization (https://www.nature.com/collections/organomediated-polymerization), which showcases latest research efforts on making plastics more sustainable. The publication highlighted here reports the depolymerization of a super engineering plastic, PEEK, from pellets, films, or even carbon- or glass fiber-enforced material into monomers that in turn can be re-polymerized.”

Chief Editor Dr Victoria Richards highlights *In situ micropillar compression of an anisotropic metal-organic framework single crystal* by Jin-Chong Tan et al. (10.1038/s42004-023-00858-w). “In this contribution, the authors describe an experimental method to characterize the anisotropic mechanical properties of metal-organic frameworks, by creating micropillars, compressing them in situ, and subsequently analyzing the stress-strain curves. The team demonstrate this approach using HKUST-1, obtaining previously unavailable information on the elastic and plastic responses of this well-studied MOF.”

Editorial Board Member Prof. Kristin Wustholz highlights *Near-infrared hyperspectral imaging to map collagen content in prehistoric bones for radiocarbon dating* by Sahra Talamo et al. (10.1038/s42004-023-00848-y). “The team uses near-infrared (NIR) hyperspectral imaging to generate chemical maps of collagen in ancient bones. This non-destructive and non-invasive technique can be used as a precursor step to determine which bone samples are viable for destructive radiocarbon dating, thereby preventing the unnecessary destruction of precious and protected ancient bones.”

Editorial Board Member Dr David Nelson highlights *Tetramethylphosphinane as a new secondary phosphine synthon* by Martin van Meurs et al. (10.1038/s42004-023-00876-8). “This paper draws inspiration from the application of bulky bases derived from 2,2,6,6-tetramethylpiperidine (TMP) and details the synthesis of the phosphine analogue. Bulky phosphine ligands often lead to catalysts with useful reactivity in a range of reactions, so the development of a scalable and practical synthetic route to this new scaffold is potentially very valuable. I look forward to seeing what this team, and others in the field, achieve using this new ligand.”

Editorial Board Member Dr Indranath Chakraborty highlights *Photoinduced edge-specific nanoparticle decoration of two-dimensional tungsten diselenide nanoribbons* by Aleksandar Matkovic et al. (10.1038/s42004-023-00975-6). “Integration of metal nanoparticles on 2D materials is often restricted to either the top or bottom of the 2D flakes. Here, the authors introduced an approach for edge-specific nanoparticle decoration via light-assisted reduction of silver ions followed by merging silver seeds. Arrays of the self-limited in size silver nanoparticles were seen along the tungsten diselenide WSe_2_ nanoribbon edges. The density of nanoparticles is tunable by external stimuli such as laser fluence.”

Editorial Board Member Dr Satoshi Honda highlights *Real-time* ^*31*^*P NMR reveals different gradient strengths in polyphosphoester copolymers as potential MRI-traceable nanomaterials* by Frederik Wurm et al. (10.1038/s42004-023-00954-x). “The team has synthesized various polyphosphoester copolymers and terpolymers based on organocatalytic ring opening polymerization and has succeeded in analyzing the polymerization kinetics with real-time ^31^P NMR. Encouraged by the MRI-traceability and potential biodegradability of the developed polymers, the research could facilitate the development of nanomaterials for bio-related applications.”

Editorial Board Member Prof. Andy Wilson highlights *Species-specific lipophilicities of fluorinated diketones in complex equilibria systems and their potential as multifaceted reversible covalent warheads* by Yossi Zafrani et al. (10.1038/s42004-023-01004-2). “This is a really nice thorough manuscript that applies physical organic chemistry principles, DFT calculations and state-of-the-art NMR methods to a development of warheads for chemical biology applications—a major current topic in chemical biology. The work assesses fluorinated diketones (FDKs) and their equilibria in the presence of nucleophiles. A crucial consideration is the lipophilicity of the warheads and the resultant products arising from reaction with nucleophiles. Using a novel ^19^F-NMR method to determine log P established that reaction leads, in some cases, to lipophilic to hydrophilic shifts, which is key for adapting to different environments. Reaction of the CF_2_(CO)_2_ warheads with cysteine residues resulted in different regiochemical outcomes, further broadening the potential scope of these warheads for development of covalent drugs.”

Associate Editor Dr Huijuan Guo highlights *Experimental phasing opportunities for macromolecular crystallography at very long wavelengths* by Armin Wagner and collaborators (10.1038/s42004-023-01014-0). “The team used the long-wavelength beamline I23 at Diamond Light Source to extend the accessible wavelength range for macromolecular crystallography to *λ* = 5.9 Å, accessing the absorption edges of biologically important lighter atoms such as calcium, potassium, chlorine, sulfur, and phosphorous by native-single-wavelength anomalous diffraction techniques. This work implies that long-wavelength crystallography is a compelling option for experimental phasing.”

Editorial Board Member Prof. Hind Al-Abadleh highlights *Unraveling surface and bulk dynamics of iron(III) molybdate during oxidative dehydrogenation using operando and transient spectroscopies* by Christian Hess et al. (10.1038/s42004-023-01028-8). “The team used a combination of techniques that include operando Raman and impedance spectroscopy combined with transient IR spectroscopy and X-ray photoelectron spectroscopy to investigate the contributions of surface active sites and atomic diffusion within the bulk to the mechanism of propane oxidative dehydrogenation. They studied this reaction as a function of temperature from 25 to 550 °C. The team measured directly and under reaction conditions the presence of a molybdenum-rich surface layer in Fe_2_(MoO_4_)_3_ containing Mo=O bonds responsible for the abstraction of hydrogen from C–H bonds. They also reported that “*the presence of iron greatly influences the reactivity behavior via oxygen diffusion but is moderated in its oxidative capacity by surface MoOx*.” The insights gained from the study are significant because of direct access to the fundamental properties of the iron(III) molybdate catalyst, which could be applied to other oxide materials to obtain detailed mechanistic understanding of the mode of operation of selective oxidation catalysts.”

Editorial Board Member Prof. Wei Zhang highlights *Enhanced interfacial water dissociation on a hydrated iron porphyrin single-atom catalyst in graphene* by Marie-Laure Bocquet et al. (10.1038/s42004-023-01027-9). “This article uses DFT calculations to explore the different reactivity of two Fe single atom catalyst defects in the hydrolysis desorption process, emphasizing the stronger interaction between Fe-porphyrin defects and adsorbed water molecules, as well as the higher stability of water dissociation products. In the biased simulations with an applied electric field, the deprotonation effect of Fe-porphyrin on adsorbed water molecules was also observed, indicating the advantage of Fe-porphyrin defects in deprotonation reactions compared to Fe-pyridine. On this basis, this work plays a guiding role in reasonably designing the coordination of monatomic catalysts and accelerating the process of hydrolysis and adsorption on the catalyst surface.”

Editorial Board Member Dr Per-Olof Syrén highlights *Fe protein docking transduces conformational changes to MoFe nitrogenase active site in a nucleotide-dependent manner* by Brian Bothner et al. (10.1038/s42004-023-01046-6). “The authors used millisecond time-resolved hydrogen-deuterium exchange mass spectrometry combined with computational normal mode analysis applied to MoFe nitrogenase. The results show how protein dynamics in the FeMo-co active site are effected by distal Fe protein binding, implying a role of long-range coupled protein motion for catalysis.”

Editorial Board Member Dr Teodoro Laino highlights *Modelling local and general quantum mechanical properties with attention-based pooling* by David Buterez et al. (10.1038/s42004-023-01045-7). “This research underscores ‘attention’ as a pivotal component in contemporary scientific research works, demonstrating its prowess in capturing nonlinear, localized, and diverse patterns across various properties. It highlights the transformative impact of attention mechanisms, which not only augment predictive accuracy but also enrich the expressivity of the neural network models. Used as a pooling function, the attention not only elevates model performance but also remains adaptable across diverse datasets, without imposing significant computational overhead. The findings not only endorse its efficacy in predicting intensive, localized properties like HOMO and LUMO energies, but also reveal its unexpected performance enhancement in broader properties, revolutionizing the realm of data-driven quantum modeling.”

Editorial Board Member Dr Christian Agatemor highlights *Cell-compatible isotonic freezing media enabled by thermo-responsive osmolyte-adsorption/exclusion polymer matrices* by Kosuke Kuroda et al. (10.1038/s42004-023-01061-7). “Cryopreservation of cells is critical to advancing medicine and biotechnology. However, state-of-the-art cryopreservation technologies significantly damage cells by dehydration or impacting high osmotic pressure on cells. Kuroda et al. designed a poly(zwitterion)-based cryopreservation medium that functions under isotonic conditions and controls osmotic pressure by adsorbing and desorbing NaCl, successfully cryopreserving freeze-vulnerable cells.”

Editorial Board Member Prof Chris Li highlights *In operando NMR investigations of the aqueous electrolyte chemistry during electrolytic CO*_*2*_ *reduction* by Sven Jovanovic et al. (10.1038/s42004-023-01065-3). “The role of electrolytes can have a significant impact on the reaction mechanism of an electrocatalytic reaction. In this study, the authors report an operando NMR technique to investigate the HCO_3_^-^ electrolyte for the CO_2_ electrochemical reduction reaction. It was found that HCO_3_^-^ exists either as a free ion or as a solvent-ion pair under different conditions. The dynamic equilibrium between these two states of the electrolyte will affect the resupply rate to the CO_2_ electrolysis.”

In addition to the above highlighted primary research Articles, we are pleased to include within our Editors’ Highlights Collection a number of timely and informative Review and Perspective pieces, as well as valuable opinion pieces from the community in the form of Comment articles. We hope you enjoy browsing this Collection of exciting contributions to *Communications Chemistry*.

